# Mucosal leak after peroral endoscopic myotomy: what to do?

**DOI:** 10.1055/a-2446-6638

**Published:** 2024-11-08

**Authors:** Francesco Cocomazzi, Marco Gentile, Sonia Carparelli, Laura Varano, Francesco Perri

**Affiliations:** 1 577188 Division of Gastroenterology and Digestive Endoscopy, Fondazione Casa Sollievo della Sofferenza IRCCS, San Giovanni Rotondo, Italy; 2 577188 Anesthesiology and Intensive Care Medicine, Fondazione Casa Sollievo della Sofferenza IRCCS, San Giovanni Rotondo, Italy

Peroral endoscopic myotomy (POEM) is becoming the first-line therapy for achalasia. It is a safe and minimally invasive treatment with a low rate of adverse events (AEs). The integrity of the mucosal flap at the end of POEM and adequate closure of the incision site are essential to avoiding AEs 1 2 3 4 5 . Postoperative AEs (e.g. leaks, late mucosal perforations) are considered difficult to diagnose and manage 4 .

An 84-year-old man with type II achalasia underwent POEM, using an anterior approach ( Video 1 ). The following day, an esophageal X-ray with water-soluble contrast showed a double-lumen appearance ( Fig. 1 ). An esophagogastroduodenoscopy revealed leakage distal to a dehiscent mucosotomy ( Fig. 2 ). After an unsuccessful attempt to close the leak with endoclips, we placed a fully covered metal stent (ESP1808F; TaeWoong Medical, Gimpo, South Korea) as described in the literature 1 2 . A subsequent computed tomography scan showed no collections or abscesses but residual contrast in the false lumen ( Fig. 3 **a, b** ). Through a literature search, we found the paper by Familiari et al. 3 , and 2 days after stent placement we removed the endoprosthesis. Using a Triangle-Tip J Knife (Olympus, Tokyo, Japan), we incised the mucosal flap with EndoCut current (Erbe Elektromedizin GmbH, Tübingen, Germany), without complications. After 48 hours, the scar showed a regular appearance, esophagogram showed no leakages ( Fig. 4 ), and the patient resumed oral feeding without complications. One month later, the mucosa had almost re-epithelialized ( Fig. 5 ).

**Fig. 1:**
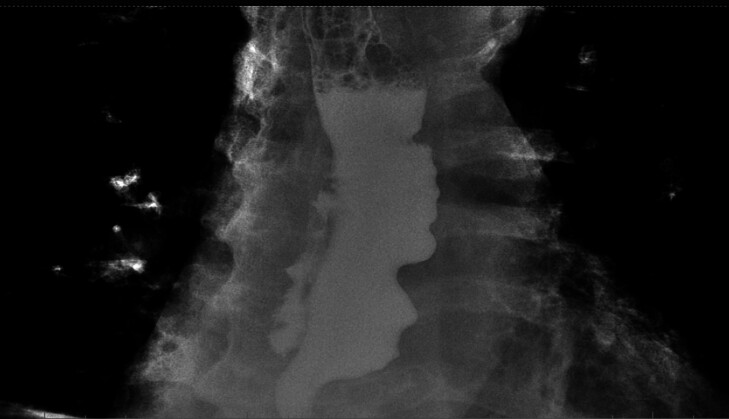
Esophageal X-ray with water-soluble contrast 24 hours after peroral endoscopic myotomy: esophageal double-lumen appearance.

**Fig. 2:**
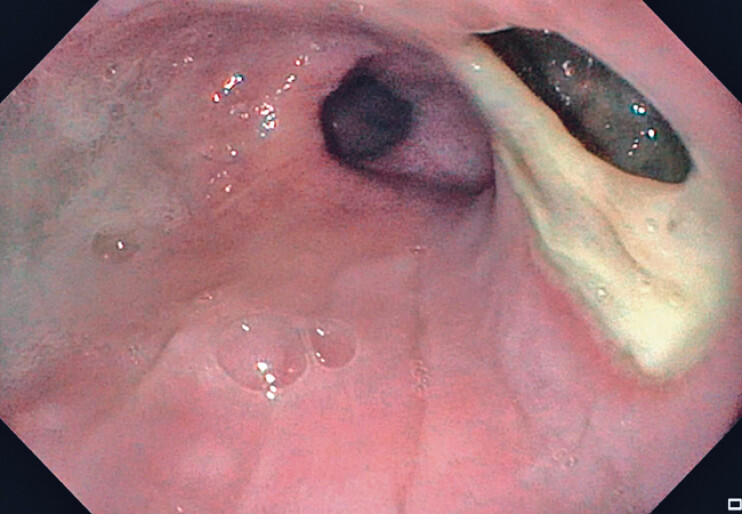
Large leakage distal to the dehiscent mucosotomy site.

**Fig. 3:**
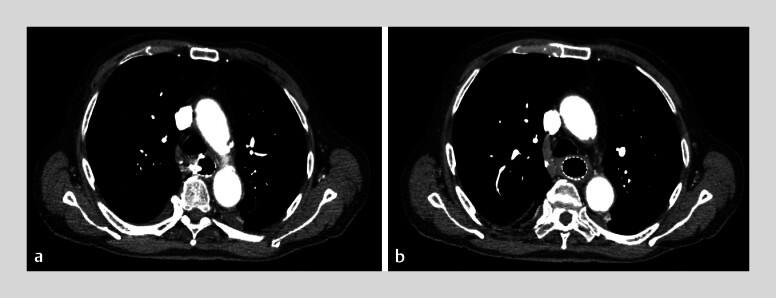
Computed tomography. **a, b** Features after stent placement.

**Fig. 4:**
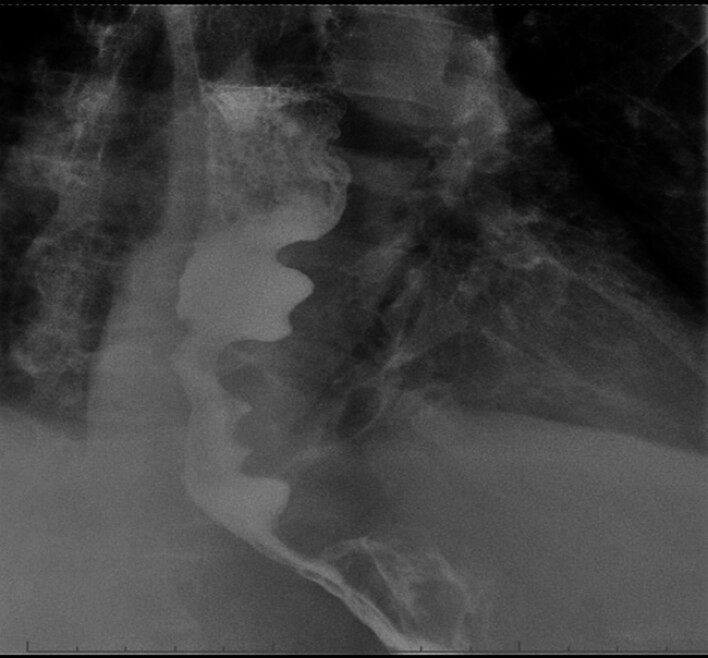
Esophagogram features after flap mucosotomy.

**Fig. 5:**
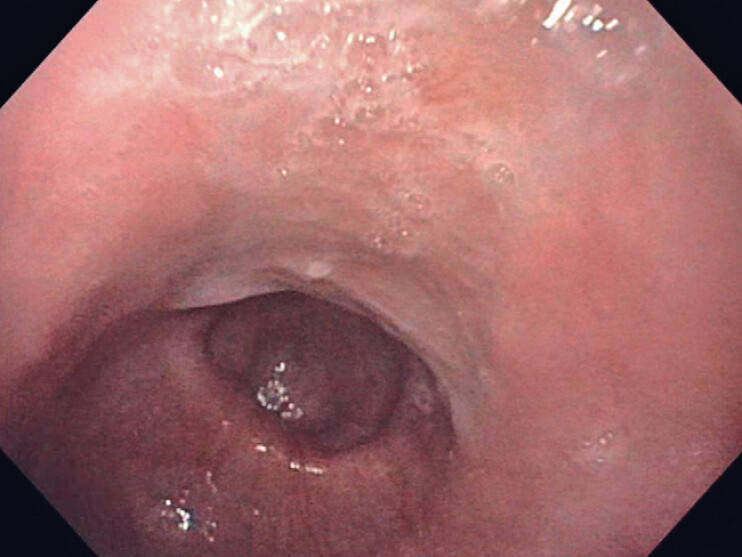
Scar features 1 month later.

Management of a mucosal leak after peroral endoscopic myotomy.Video 1

Unlike other cases 2 3 , our complication was detected early, in an asymptomatic patient (although he had only resumed drinking). Furthermore, the mucosotomy performed 4 days after POEM has proven to be a safe and effective choice to manage this complication.

To date, performing diagnostic examinations to exclude AEs is not required in stable patients 1 . Nevertheless, given that the complication described here was in an asymptomatic patient, it is natural to wonder whether some patients would benefit from performing diagnostic tests before refeeding.

Endoscopy_UCTN_Code_TTT_1AO_2AP

## Notes

Endoscopy E-Videos https://eref.thieme.de/e-videos
*E-Videos* is an open access online section of the journal *Endoscopy* , reporting on interesting cases and new techniques in gastroenterological endoscopy. All papers include a high-quality video and are published with a Creative Commons CC-BY license. Endoscopy E-Videos qualify for HINARI discounts and waivers and eligibility is automatically checked during the submission process. We grant 100% waivers to articles whose corresponding authors are based in Group A countries and 50% waivers to those who are based in Group B countries as classified by Research4Life (see: https://www.research4life.org/access/eligibility/ ). This section has its own submission website at https://mc.manuscriptcentral.com/e-videos .
